# Numerical Simulation of Optical Sensing by the Far Field Pattern Radiated by Periodic Grating Strips Over Silica Buffer on the Silicon Wire Waveguide

**DOI:** 10.3390/s20185306

**Published:** 2020-09-16

**Authors:** Andrei Tsarev, Vittorio M. N. Passaro

**Affiliations:** 1 Laboratory of Optical Materials and Structures, Rzhanov Institute of Semiconductor Physics, SB RAS, 630090 Novosibirsk, Russia; tsarev@isp.nsc.ru; 2 Laboratory of Semiconductor and Dielectric Materials, Physics Department, Novosibirsk State University, 630090 Novosibirsk, Russia; 3 Photonics Research Group, Dipartimento di Ingegneria Elettrica e dell’Informazione, Politecnico di Bari, Via E. Orabona 4, 70125 Bari, Italy

**Keywords:** optical sensors, silicon wire, silicon oxynitride (SiON), segmented grating, far field pattern, numerical modeling, finite difference time domain (FDTD) method

## Abstract

This paper presents results of numerical modeling of a modified design of an optical sensor based on segmented periodic silicon oxynitride (SiON) grating evanescently coupled with silicon wire. This segmented grating works as a leaky waveguide, which filters input power from a broadband optical source and radiates it as an outcoming optical beam with both a small wavelength band and a small beam divergence. The radiation angle strongly depends on the refractive index of the grating environment and provides sensor interrogation by measuring the far field pattern in the focal plane of the lens, which is placed near the sensor element. The device concept was verified by direct numerical modeling through the finite difference time domain (FDTD) method and provided moderate intrinsic limit of detection (iLOD) ~ 0.004 RIU with a possible iLOD ~ 0.001 RIU for 10 mm-long structures.

## 1. Introduction

Publications on optical sensors cover about 20% [1] of all publications registered by the Web of Science. These sensors utilize different arrangements [1,2,3,4,5,6,7,8,9,10,11,12,13,14,15] for reading signals. Among them, a lot of optical sensors use wavelength interrogation based on high precision measuring of the shift in resonance wavelength in the photonic structure, and the value depends on the environment of the sensor element [1,2,3,4,5,6,7,8,9,10]. Wavelength interrogation can be accomplished using a tunable laser with a fine linewidth or by launching the optical beam into the waveguide, which contains a broad wavelength spectrum, say, from the super-luminescence diode, and measuring the transmitting signal by the optical spectrum analyzer (OSA). Both these variants provide the possibility to develop advanced optical sensors [1,2,3,4,5,6,7,8,9,10] with the sensitivity depending on the design of the optical element and the spectrum resolution of the tunable laser or the OSA. Both readout schemes are rather costly, which prevents the wide spread of these sensor technologies. Alternative interrogation technology provides grating assisted sensors [13,14,15], which measures the far field pattern. as the radiation angle, due to Bragg conditions, is strongly dependent on optical wavelength, grating period, and environment index.

In the current paper, we present the numerical study of the modified variant of the far field pattern optical sensor with the segmented grating. The periodic segmented structure works both as the pass band filter, which selects part of the spectrum energy from the broadband input optical source, and as the grating coupler, which radiates power to be focused by the lens on the multi-element photonic sensor, which is used to measure the far field pattern. The previous variant of such a sensor [16] was based on the optical phenomena of “abnormal blocking” [17,18], which takes place in the silicon wire with the periodic segmented grating coupled by the evanescent field through the silica buffer. This design provides very high sensitivity (~400 nm/RIU) but unfortunately has very low intrinsic limit of detection (iLOD) ~ 0.01 RIU due to high propagation loss of the leaky waveguide formed by long-period segmented grating. The idea of this paper is to present the modified sensor design, which has both high sensitivity and moderate iLOD, which is useful for application as the liquid sensor works in water and has a high optical loss in the telecom band.

## 2. Sensor Design

**Figure 1:**
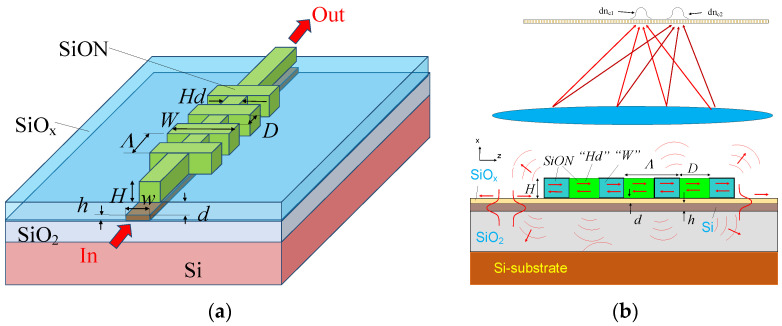
The scheme of the optical sensor based on the interaction of the guided wave with the virtual leaky wave of the segmented grating. (**a**) General view of segmented structure; (**b**) general arrangement of far field interrogation: optical waves corresponding to the different index increments (dn_c1_ and dn_c2_) were focused at different positions on the photodetector screen.

The structure design, which is under investigation, is shown in Figure 1. The main reason for the high loss in the long-period grating leaky waveguide [17,18,19] is the radiative scattering of the diverge wave propagating in the free space of the environmental sensor between the segments. One can decrease this loss by decreasing the gap D (<200 nm), but it provides some disadvantage due to effect of the partial gap filling by the liquid [20], which will course some degradation of the sensor performances.

We propose the use of segmented structures with a wide gap (D > 1000 nm) by implementing the modified design that includes close-connected strips between the segments (see Figure 1a). The strip width Hd = 450 nm was close to the fundamental mode cutoff. From one side, it did not cause significant degradation of sensitivity related to pure segmented grating, but from the other side, the optical wave that left the segmented part was guided forward up to the next segment and thus decreased the propagation loss in the structure.

Note that this design is very similar to the conventional grating filter, with the rectangular grating manufactured on the waveguide sides [21]. However, this grating filter was optimized for sensor application. Thus, contrary to the known design of the grating filter, this grating accomplished the two-stage process: at first, it provided coupling from the guide to the leaky modes, then this leaky wave radiated power by the same grating during the propagation along the structure. This radiated wave was focused by an additional lens on the multi-element detector, such as the CCD (charge coupled device) camera placed on the focal plane (see Figure 1b). This lens was placed over the flow chambers (not shown) and provided the possibility to measure and analyze the far field pattern variation under an index change of the liquid index under investigation.

The silicon wire had the height h = 250 nm and the width w = 450 nm. It was placed on a thick 2 µm silicon dioxide (n_b_ = 1.444) buffer. It corresponded to the optical waveguide, which supported the single quasi-TE (transverse electric)-polarized guided mode (electric field in the plane of the waveguide). The segmented structure was achieved by the periodic (Λ = 1.45 µm) strips from silicon oxynitride (SiON), with the index at n_p_ = 1.80. The desired value index of SiON can be given by the composition ratio X_SiO2_ = 0.653 of SiO_2_ in the SiON:

n_pSiON_ = N_SiO2_ + X_SiO2_·(N_Si3N4_ − N_SiO2_) (1)

The segmented grating strip length D_s_ = Λ − D was controlled by the period Λ and the etching gap D. We used the condition D = 0.2 µm, which provided the compromise between the sensitivity and the propagation loss. The typical height and width were H = 1.0 µm and W = 1.0 µm, respectively. Grating was separated from the silicon waveguide by a silica overlayer, with height d = 400 nm and index n_s_ = 1.4.

In the current sensor design, the guided optical beam, which contained a broad spectral range, arrived from the left into the grating area (see Figure 1a). The presence of the periodic grating provided resonance-type interaction (at optical wavelength λ_m_) of the guided mode in the silicon wire (effective index N_g_) and the leaky mode (effective index N_L_), propagated in the segmented SiON structure. The full width at half-maximum (FWHM) of the grating resonance Δλ was mainly limited by the structure length and propagation loss of the leaky wave and typically was in the range of 1–5 nm. This filtered part of the input spectrum was coupled into the segmented structure and then was radiated along the propagation. The radiated angle of the maximum intensity at optical wavelength λ_m_ was described by the Bragg condition and provided information about the index of the environment n_c_.

The fabrication of such structures could be carried out by using a silicon photonic process developed within the conventional CMOS (complementary metal oxide semiconductor) foundry. For example, one can use the technology already used to manufacture a spot-size converter (SSC) with a SiO_2_ spacer layer between tapered Si and SiON waveguides for fiber-to-chip coupling [22]. They deposited SiO_2_ and SiON with a low refractive index at a low temperature by the PECVD (plasma enhanced chemical vapor deposition) method. For the SSC with the SiO_2_ spacer, the SiO_2_ (n = 1.45) spacer layer was deposited on the Si wire waveguide at 350 °C, followed by deposition of a 1.2-μm-thick SiON (n = 1.62) film from a gas mixture of N_2_O, NH_3_, and SiH_4_ at 300 °C. A 1.2-μm-square SiON waveguide with vertical walls was fabricated with an i-line stepper and by dry etching with CHF_3_ (trifluoromethane). It must be noted that the fabricated SiON waveguide cores are somewhat distorted and are related to the rectangular shape as the thin SiO_2_ layer is not planarized when it covers the silicon wire. That is not important for SSC, but in our modeling, we assumed that structures with a planarized SiO_2_ spacer were inserted between the Si wire waveguide and the SiON secondary waveguide. The flat structures could be manufactured by using flowable oxide (n = 1.40) or by introducing the lift-off process used for the planarization of the waveguide surface similar to technology described in [23], which used Cr as the mask for the etching of the core and the cladding materials for TiO_2_/Ta_2_O_5_/SiO_2_ structure. In our case, the Cr mask could be used to develop the silicon wire. A SiO_2_ thin layer (equal to the height of the silicon wire) could then be deposited by the PECVD method over the silicon wire waveguide. Next, the SiO_2_ layer on the Cr mask was lifted off by wet etching of Cr. Thus, a flat surface was obtained. After planarization of waveguide surface, one can follow all of the technology process used to manufacture a spot-size converter [22], with the only difference found in the mask shape for the SiON waveguide, which now contains the grating strips.

## 3. Numerical Modeling

This structure is investigated by numerical modeling, which has been performed using the finite difference time domain (FDTD) method, which has proved its reliability many times for such a task [24]. For simulation, we utilized the commercial software package FullWave by RSoft-SYNOPSYS [25]. In order to examine the long structures, we utilized FDTD under two-dimensional (2D) approximation based on the effective index method (EIM) [26].

As we have discussed before, the EIM has a fundamental limitation [27,28], which takes place when trying to analyze the pulsed excitation of waveguide structures by the 2D FDTD method. The reason is that, in this case, the two-dimensional EIM approximation does not take into account the waveguide dispersion and thus considers the wrong value of the group index [27], which determines both the sensitivity and the linewidth. The typical error could be as much as 40% or more [27].

We propose the dispersion compensated algorithm [17] to fix this error problem. The presence of waveguide dispersion leads to the fact that the resonance wavelength λ_m_, which is determined by the 2D FDTD modeling, depends on the center wavelength λ_0_, at which the impulse excitation and the spectrum analysis of the waveguide structure is carried out. It was shown [17] that the correct drop wavelength could be found from the condition λ_m_(λ_0_) = λ_0_, which provides λ_m_ to be exactly equal to the desired condition obtained for the monochromatic excitation of a waveguide. As a result of this description, the correct wavelength responses at any desired dn_c_ corresponded to those simulations, when λ_m_(λ_0_) = λ_0_. For the simplicity, we determined two basic wavelengths λ_m_ for two cases, dn_c_ = 0 and dn_c_ = 0.01. We then implemented the linear interpolation for the wavelength coordinate:

λ_c_ = A + B·λ_0_ (2)

where A = 446.4 nm and B = 0.7112, which are obtained from these two basic wavelength simulations. Thus, all wavelength responses of the transmitting power to the leaky wave T_2_(λ_c_) will now correctly take into account the waveguide dispersion, which provides much more accuracy in the determination of all sensor parameters, including the iLOD and sensitivity S_n_. We called this algorithm the dispersion compensated effective index method (DCEIM). One must note that this method can be applied for any numerical tasks that implement the 2D FDTD method to examine the impulse propagation in the waveguide structures.

Using this dispersion compensated algorithm, we found, using the 2D FDTD method, the sensitivity S_n_ = ∂λ/∂n_c_ = 340 nm/RIU (refractive index unit) for the wavelength interrogation. Note that the similar modeling with traditional EIM provides strongly incorrect values for the sensitivity S_n_ = 480 nm/RIU.

**Figure 2:**
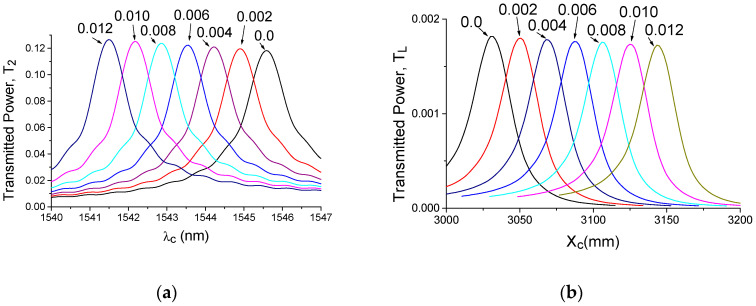
Simulated characteristics of the optical sensors and different index variations dn_c_ of the environment (water). (**a**) Transmitting power (T_2_) relative to the input signal of the leaky wave propagated in segmented grating; (**b**) the total power (T_L_) distribution (as a sum of 100 different wavelengths) in the focal plane of the ideal lens with 10 mm focal length. Simulation by the two-dimensional (2D) finite difference time domain (FDTD) under the dispersion compensated the effective index method approximation, which included the linear transformation of the coordinates (2) and (3).

As mentioned before, the sensor element based on the segmented grating over the silicon waveguide can implement different readout schemes. The application of the DCEIM to traditional wavelength interrogation is illustrated in Figure 2a. This application is typical for a lot of sensors but it requires tunable lasers or a high resolution OSA. For the far field interrogation, it is possible to measure the cross-field distribution on the focal plane of the lens (see Figure 2b). To improve the accuracy, we also implemented the DCEIM algorithm, which, in our case, included the linear transformation of the coordinate according to the relation:

X_c_ = A_x_ + B_x_·X (3)

where A_x_ = 156.5 and B_x_ = 0.948, which are obtained from linear interpolation of the simulation results for dn_c_ = 0 and dn_c_ = 0.01.

For the maximum number of segments (M = 1024), which were possible to simulate in our case, we got almost the same value of intrinsic limit of detection for both the wavelength interrogation (iLOD_w_ = δn·Δλ/Δλ_FWHM_ = 0.0038 RIU) and the far field interrogation (iLOD_FF_ = δn·ΔX/ΔX_FWHM_ = 0.0033 RIU). Here, for Δλ ·and ΔX, the shift of the peaks position for the index increment δn, Δλ_FWHM_, and ΔX_FWHM_ were the relative full width at half-maximum.

**Figure 3:**
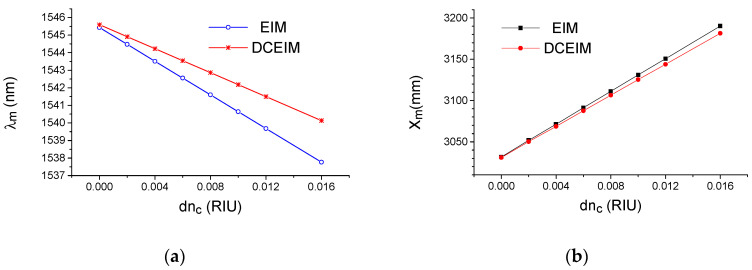
Simulated characteristics of the optical sensors obtained under the effective index method (EIM) and the dispersion compensated effective index method (DCEIM) approximations for the different index variations dn_c_ of the environment (water). (**a**) Wavelength position λ_m_ of the maximum transmitting power to the leaky wave propagated in segmented grating; (**b**) the coordinate position X_m_ of the maximum transmitting power in the focal plane of the ideal lens with 10 mm focal length. Simulation by the 2D FDTD.

The extremum positions, λ_m_ and X_m_, as a function of the different index variations dn_c_ of the environment (water) for the wavelength and far field interrogation, respectively, are shown on Figure 3. One can see their linear dependences on dn_c_ with the constant slope, which describes the sensitivity of the sensor. For example, the dispersion compensated algorithm provides the sensitivity S_n_ = ∂λ/∂n_c_ = 340 nm/RIU for the wavelength interrogation. However, we have to highlight that similar modeling under the traditional EIM provides a strongly incorrect value for the sensitivity S_n_ = 480 nm/RIU.

It must be noted that by increasing the grating length (L_g_ = Λ × M), one can increase, to some extent, the wavelength resolution, but soon it will come to the maximum value restricted by the loss of the leaky way. For example, a long sensor will reach iLOD ~ 1.5 × 10^−3^ RIU (L_g_ = 5 mm) and iLOD ~ 1 × 10^−3^ RIU (L_g_ = 10 mm). We estimated the minimum possible iLOD by analyzing the short structure (M = 3) by using the periodic boundary conditions. It was found that the minimum iLOD for our design was around 7.6 × 10^−4^ RIU and it occurred due to the high optical loss in water at the optical wavelength (1550 nm).

Note that the measuring arrangement was then much cheaper than for the case of the optical spectrum analyzer (OSA) or the tunable laser. The experimental limit of detection (LOD) depends on the experimental setup, integration time, temperature fluctuations, and the noise of the system, which is limited by intensity fluctuations [29]. Typically, it is possible to measure the wavelength position with an accuracy of about 1/15 of the FWHM [30]. Thus, we got the experimental LOD = iLOD/15 < 0.0001 RIU. For the case of the far field interrogation, the fluctuations of the intensity at different wavelengths had to be averaged within the angle divergence of the monochromatic wave. The pattern distribution measured at the focal plane of the lens (see Figure 2b) was much smoother than the relative wavelength response measured by the OSA. As the smaller intensity fluctuations were important for decreasing the LOD [29], thus the experimental LOD of our far field interrogation design had to be much smaller than for the wavelength interrogation, with a broad optical source of the same structure. This makes it possible to determine a small variation of the refractive index (below 0.0001 RIU) by a very simple and cheap interrogation scheme.

Note that, in our design, we proposed to use a cheap broadband optical source instead of a costly tunable laser or OSA. Part of the input energy spectrum was selected by the grating coupler, which was further diffracted by the same grating. The angle divergence, when projected on the focal plane of the lens, was always larger than the monochromatic illumination of the same structure by the tunable laser with a small line width (as with the conventional grating sensor [13].) Thus, in any case, the experimental LOD of the proposed sensor is larger than the competitor that utilizes the tunable laser with a linewidth smaller than the sensor linewidth. Our design by the expense of some degradation of the sensitivity is intended to use the cheaper components (say, no tunable laser or OSA) and to provide the possibility of broad use of it as a liquid type of sensor, where the experimental LOD (due to high optical loss in water) is several orders larger than for the case of gas sensors used by other structures [31], such as photonic crystals [32] or phase-shifted Bragg gratings [33] with super high quality factors.

## 4. Conclusions

We have described and carried out numerical modeling of a modified type of optical sensor with far field interrogation, which utilizes the segmented periodic structure with narrow connected strips placed over silica buffer on the silicon wire. The tunnel coupling of the guided wave to the segmented structure provides excitation of the leaky wave in the periodic segmented grating. The latter radiates power as an outgoing optical beam. This process has a resonance nature and takes place at a small wavelength range (around 1 nm), thus the outgoing optical beam has a small divergence, which makes it possible to determine small index variations of environmental liquid by measuring the shift in the far field pattern position. This can be accomplished by commercial CCD arrays placed in the focal plane of the lens.

The sensor resolution is quite high (iLOD = 0.001–0.004 nm/RIU), and, by the internal smoothing of the radiated pattern, one can get the experimental LOD smaller than 0.0001 RIU. Additionally, the sensor design is simpler and cheaper than other state-of-the-art optical solutions, where the sensor resolution depends on a more expensive design based on the use of the tunable laser or OSA.
